# A Case of Peutz-Jeghers Syndrome Presenting With an Inoperable Periampullary Tumor

**DOI:** 10.7759/cureus.14359

**Published:** 2021-04-08

**Authors:** Muhammer Ergenç, Tevfik Kıvılcım Uprak

**Affiliations:** 1 General Surgery, Istanbul Sultanbeyli State Hospital, Istanbul, TUR; 2 General Surgery, Marmara University School of Medicine, Istanbul, TUR

**Keywords:** peutz-jeghers syndrome, periampullary tumors, gastric outlet obstruction

## Abstract

Peutz-Jeghers syndrome (PJS) is a syndrome characterized by multiple hamartomatous polyps in the gastrointestinal system and melanin pigments accumulating in the skin and mucous membranes. Patients with PJS have an increased risk of gastrointestinal malignancies. In this syndrome, pancreatic cancer is primarily detected in older ages. In this article, we present a case of a patient with an unresectable periampullary tumor and PJS.

## Introduction

Peutz-Jeghers syndrome (PJS) is a syndrome characterized by multiple hamartomatous polyps in the gastrointestinal system and melanin pigments accumulating in the skin and mucous membranes [1]. Patients with PJS have an increased risk of gastrointestinal and non-gastrointestinal malignancies. The lifelong risk of gastrointestinal cancer in individuals with PJS is 38%-66% and is relatively higher compared to a healthy population [2]. The risk of developing pancreatic cancer increases with age. It can increase from 3% at the age of 40 to 11% at 70 [3]. This report is about a patient who presented with a periampullary tumor, and PJS detected who was admitted with symptoms of gastric outlet obstruction.

## Case presentation

A 34-year-old female patient, who had been admitted to the emergency service complaining of weight loss and a gradual increase in nausea and vomit for two months, was hospitalized in the general surgery clinic for further evaluation.

**Figure 1:**
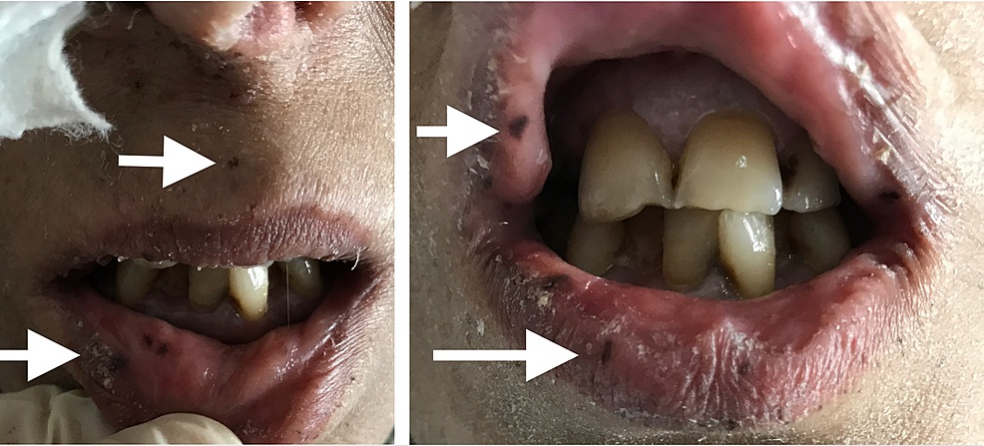
Black spots localized in the perioral area (white arrows)

Abdominal guarding or rebound tenderness was not detected in the patient's physical examination, but a palpable firm and massive lesion in the periumbilical region was found. The patient was cachectic, and perioral hyperpigmentation (black spots) was detected (Figure 1).

The patient had an abdominal surgery about 25 years ago but did not know the indication. There was no history of prior illness or medication. Her patient body mass index was 16.9 kg/m^2^.

Laboratory tests revealed a white blood cell count of 14.2 x 10^3^/µL, normocytic anemia with a hemoglobin level of 10.6 g/dL, albumin 3.6 g/dL, total protein 7.8 g/dL, aspartate aminotransferase 35 U/L, alanine aminotransferase 30 U/L and total/direct bilirubin 1.11/0.35 mg/dL.

**Figure 2:**
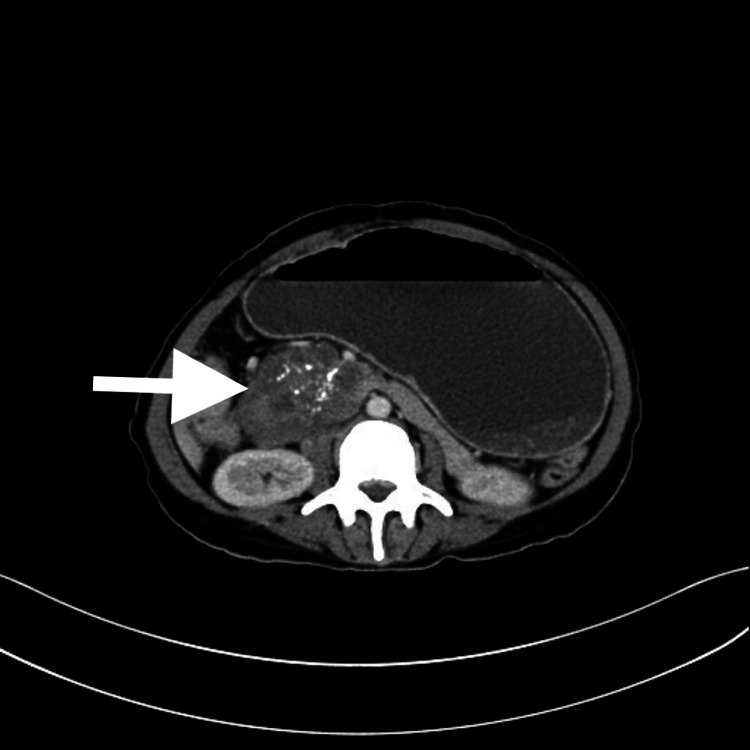
Axial image of the abdomen CT scan showing a mass in the head of the pancreas (white arrow)

**Figure 3:**
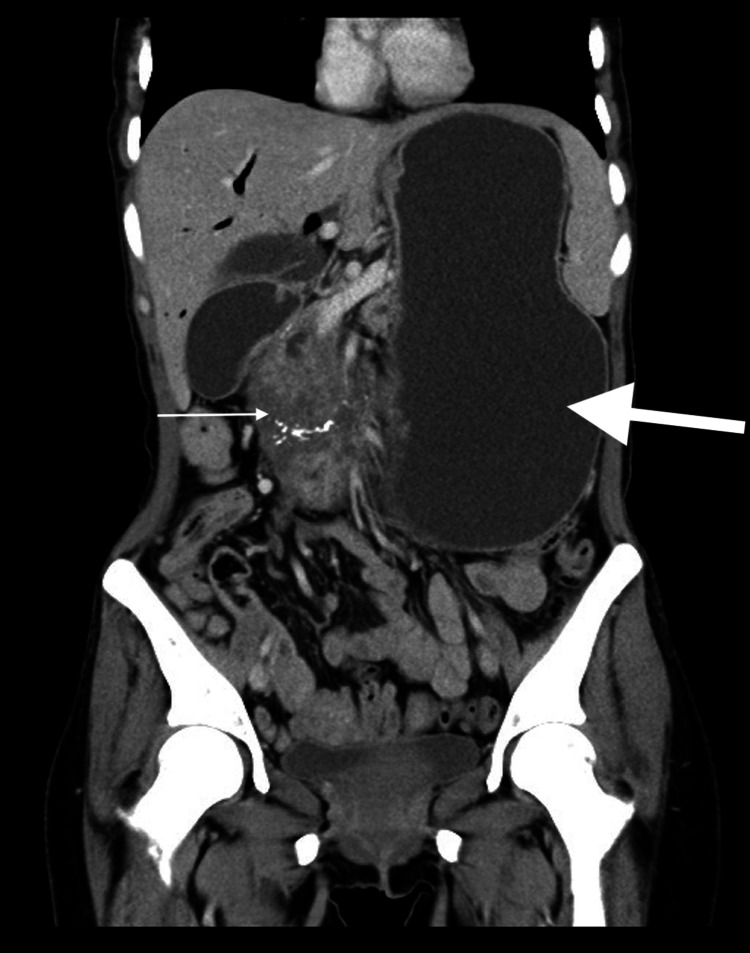
Coronal reformat image of the abdomen CT scan showing a mass in the head of the pancreas (thin arrow) and massive gastric dilatation (thick arrow)

Intravenous contrast-enhanced abdominal tomography showed a heterogeneous mass of approximately 10 x 7 x 5 cm at the pancreatic head level. The mass had cystic areas and calcification. It was observed that the stomach was massively dilated due to the compression of the duodenal mass, and air in the intrahepatic bile ducts was detected (Figures 2-3) [4].

Multiple polypoid lesions were detected in the gastric fundus in the upper digestive endoscopy. The pylorus could not be passed due to post-pyloric obstruction.

Explorative laparotomy was performed. The tumoral mass in the head of the pancreas invaded the retroperitoneal area, and the peritumoral structure was found. Peritoneal tumors were observed on the gallbladder and hepatoduodenal ligament. Multiple intestinal invaginations were observed, and intestinal polyps were palpated. Polyps were excised by performing multiple enterotomies. Gastrojejunostomy was also performed to resolve duodenal obstruction. Multiple excisional biopsies were obtained from the peritoneal implants. The patient was started on a liquid diet on the second postoperative day and had a passage of flatus on the third day. On the fourth postoperative day, the patient was allowed to eat a solid meal. She was discharged on the fifth postoperative day. Pathological examinations of peritoneal tumors were compatible with mucinous adenocarcinoma, and small bowel polyps were reported as hamartomatous lesions (smooth muscle structure branching into thick bundles was observed). The patient was diagnosed as a stage 4 periampullary tumor due to metastatic peritoneal implants and referred to the medical oncology and medical genetics departments for treatment and PJS family screening. The patient died within six months of a diagnosis.

## Discussion

PJS is an autosomal dominant genetic disease caused by a germline mutation in the STK11 (LKB1) gene. The incidence of PJS is approximately between 1/50,000 and 1/200,000 (live birth) [5]. The average age for PJS diagnosis is 24.3 years, and its prevalence is equal in both genders.

The most common admission symptoms of PJS are recurrent abdominal pain secondary to intussusception, anemia due to occult gastrointestinal bleeding, melena, and hematochezia. Hematemesis may occur in patients with gastric or duodenal polyps, and prolapse may occur in rectal polyps [6].

In this case, the patient was investigated with a preliminary diagnosis of gastric outlet obstruction. A mass was found in the head of the pancreas. The diagnosis of PJS was made with multiple hamartomatous polyps and mucocutaneous characteristic skin pigmentations [7]. The presentation of patients with PJS usually occurs in the second or third decade with symptoms secondary to abdominal pain and gastrointestinal polyps [8]. The risk of developing cancer in patients with PJS diagnosed at the ages of 20-30 years is 2%-5%. In 70s, the cumulative cancer risk increases approximately four times compared to the average population and reaches 85%. The most common gastrointestinal malignancies in PJS patients are seen in the colorectal region.

The diagnosis of PJS is usually made at an early age; it is recommended that patients diagnosed with it should be taken under surveillance, and specific cancer-screening programs should be implemented [9]. Surveillance for pancreatic cancer is recommended to start at the age of 30. It is predicted that pancreatic cancers may have originated from intraductal papillary mucinous neoplasms. Screening by yearly endoscopic ultrasound and/or magnetic resonance imaging protocols is recommended for the detection of precursor lesions in PJS patients [10]. In our case, patient's diagnosis was made in the fourth decade and after cancer development. Since it was late for curative treatment, palliative treatment was performed.

## Conclusions

As in our case, patients with PJS can be diagnosed in their 30s after developing pancreatic cancer. Therefore, although rare, patients with genetic cancer syndromes should be kept in screening in case of gastrointestinal and hepatobiliary cancer.

## References

[1] Arber N, Moshkowitz M (2011). Small bowel polyposis syndromes. Curr Gastroenterol Rep.

[2] van Lier MG, Wagner A, Mathus-Vliegen EM, Kuipers EJ, Steyerberg EW, van Leerdam ME (2010). High cancer risk in Peutz-Jeghers syndrome: a systematic review and surveillance recommendations. Am J Gastroenterol.

[3] Hearle N, Schumacher V, Menko FH (2006). Frequency and spectrum of cancers in the Peutz-Jeghers syndrome. Clin Cancer Res.

[4] Yılmaz N, Özkurt H, Değirmenci H, Kahraman AD, Başak M (2004). Helical CT of pancreatic cancer; assessment of resectability before surgery. Med Bull Sisli Etfal Hosp.

[5] Beggs AD, Latchford AR, Vasen HF (2010). Peutz-Jeghers syndrome: a systematic review and recommendations for management. Gut.

[6] Calva D, Howe JR (2008). Hamartomatous polyposis syndromes. Surg Clin North Am.

[7] Lindor NM, McMaster ML, Lindor CJ, Greene MH (2008). Concise handbook of familial cancer susceptibility syndromes - second edition. J Natl Cancer Inst Monogr.

[8] Zbuk KM, Eng C (2007). Hamartomatous polyposis syndromes. Nat Clin Pract Gastroenterol Hepatol.

[9] Giardiello FM, Trimbath JD (2006). Peutz-Jeghers syndrome and management recommendations. Clin Gastroenterol Hepatol.

[10] Korsse SE, Harinck F, van Lier MG (2013). Pancreatic cancer risk in Peutz-Jeghers syndrome patients: a large cohort study and implications for surveillance. J Med Genet.

